# MIMAS 3.0 is a Multiomics Information Management and Annotation System

**DOI:** 10.1186/1471-2105-10-151

**Published:** 2009-05-18

**Authors:** Alexandre Gattiker, Leandro Hermida, Robin Liechti, Ioannis Xenarios, Olivier Collin, Jacques Rougemont, Michael Primig

**Affiliations:** 1 School of Life Sciences, Ecole Polytechnique Fédérale de Lausanne (EPFL) and Swiss Institute of Bioinformatics (SIB), CH-1015 Lausanne, Switzerland; 2 Friedrich Miescher Institute for Biomedical Research, CH-4058 Basel, Switzerland; 3 Swiss Institute of Bioinformatics, Vital-IT, CH-1015 Lausanne, Switzerland; 4 Institut de Recherche en Informatique et Systèmes Aléatoires (IRISA), Université de Rennes 1, F-35042 Rennes, France; 5 Inserm, U625, GERHM; IFR-140; Université de Rennes 1, F-35042 Rennes, France

## Abstract

**Background:**

DNA sequence integrity, mRNA concentrations and protein-DNA interactions have been subject to genome-wide analyses based on microarrays with ever increasing efficiency and reliability over the past fifteen years. However, very recently novel technologies for Ultra High-Throughput DNA Sequencing (UHTS) have been harnessed to study these phenomena with unprecedented precision. As a consequence, the extensive bioinformatics environment available for array data management, analysis, interpretation and publication must be extended to include these novel sequencing data types.

**Description:**

MIMAS was originally conceived as a simple, convenient and local Microarray Information Management and Annotation System focused on GeneChips for expression profiling studies. MIMAS 3.0 enables users to manage data from high-density oligonucleotide SNP Chips, expression arrays (both 3'UTR and tiling) and promoter arrays, BeadArrays as well as UHTS data using MIAME-compliant standardized vocabulary. Importantly, researchers can export data in MAGE-TAB format and upload them to the EBI's ArrayExpress certified data repository using a one-step procedure.

**Conclusion:**

We have vastly extended the capability of the system such that it processes the data output of six types of GeneChips (Affymetrix), two different BeadArrays for mRNA and miRNA (Illumina) and the Genome Analyzer (a popular Ultra-High Throughput DNA Sequencer, Illumina), without compromising on its flexibility and user-friendliness. MIMAS, appropriately renamed into Multiomics Information Management and Annotation System, is currently used by scientists working in approximately 50 academic laboratories and genomics platforms in Switzerland and France. MIMAS 3.0 is freely available via .

## Background

Microarrays are an essential tool for high-throughput analysis of single nucleotide polymorphisms (SNPs), DNA rearrangements, RNA concentrations, exon composition and protein-DNA interactions [1-3]. Microarray technology is based on distinct manufacturing approaches such as robotic application of double stranded DNA fragments onto glass slides (spotted arrays) [4], *in situ *synthesis of high-density oligonucleotide probes (GeneChips) [5] and bead-based systems (BeadArrays) [6]. A comprehensive set of open source and commercial bioinformatics solutions have become available over the last decade that includes certified public array data repositories in Europe, the US and Asia [7-9], a platform for anonymous peer-review of genome biological manuscripts [10] and many web-based [11] or local array data management and analysis solutions [12]. International standards for data acquisition, representation and interchange developed by the Microarray Gene Expression Data Society (MGED, [13]) include the Minimum Information About a Microarray Experiment (MIAME) guidelines [14], the MicroArray and Gene Expression (MAGE) data representation standard [15], the MAGE-TAB interchange format [16] and the MGED Ontology for microarray experiment and biological sample annotation [17]. Annotating microarray data according to MIAME guidelines and depositing them in certified repositories ArrayExpress (EBI), Gene Expression Omnibus (NCBI), and CIBEX is mandatory for publishing in most scientific journals although this policy is regrettably not always rigorously enforced [18].

Most recently, novel ultra-high throughput DNA sequencing (UHTS) technologies have been developed that enable researchers to obtain the complete genomes of model organisms and *H. sapiens *much faster and at a much lower cost than classical methods [19]. Moreover, these technologies appear also to be extremely useful for accurately measuring gene expression (RNA-Seq) [20] and protein-DNA interactions (ChIP-Seq) [21]. However, no widely accepted standard exists as yet within the community to report and archive the output of UHTS experiments. Efforts are under way in collaboration with MGED's Reporting Structure for Biological Investigations (RSBI) work group to develop a single format for annotations across multiple technologies [22] and a standard for UHTS similar to what was developed earlier for microarrays (MINSEQE) [23]. Currently, UHTS experiments are annotated using the MGED Ontology prior to submission to the European Nucleotide Archive [24].

Here we report a substantial extension of our system for consortium-wide microarray data management [25]. The novel web-accessible Multiomics Information Management and Annotation System (MIMAS 3.0) is based upon an elaborate graphical user interface (GUI) and a scalable relational database. It is designed to store manually annotated expression data from several research facilities that may be organized within a consortium. Version 3.0 has been extended to support data produced by eight different types of microarrays from the two most popular manufacturers as well as annotation and genome location data derived from sequencing experiments. Data representation was standardized according to the MAGE-TAB data exchange format and MGED Ontology. A one-step export feature creating a MAGE-TAB spreadsheet is available facilitating submission to the ArrayExpress repository. MIMAS 3.0 is freely available under the GNU license at [26].

## Construction and content

### The database model

The database model was initially constructed as generically as possible which improves knowledge representation and simplifies maintenance [25]. It was therefore not necessary to modify the model when the system was extended from supporting only GeneChip expression arrays to including seven other different array types (see Additional File 1 in [25]). The software is organized around a database-driven reflective architecture [27]. This is in contrast to a more traditional design where each particular attribute values are stored in a separate column, and specific code is needed to handle the input and storage of each attribute. In an adaptation of the Type Object design pattern [28], attribute definitions are themselves objects in the database. They are defined by their names and value type. Parameters including the default value, whether multiple values are allowed, and the list of possible values if a choice list must be presented. The software reads these definitions to generate fully dynamic web forms, and stores input values in a generic table accommodating all attribute types.

An inconvenience of such generic data storage is the relative inefficiency of queries and data retrieval. However, in our experience, the amount of annotation data is well within the capability of modern relational database management engines, and even complex searches perform almost instantaneously. If search performance were to become an issue, materialized views could easily be introduced to create a de-normalized data mart for efficient searching.

Since the MGED Ontology is limited and does not cover domains such as chemical compounds, MIMAS contains its own controlled vocabulary that greatly overlaps the MGED Ontology. This controlled vocabulary is extended in a controlled manner (via curation) via direct user input. Users can call up a list of all available annotation terms and they can select an option to enter novel terms as they deem appropriate. These terms are validated by the database curator and included into the controlled vocabulary.

### The web application

**Figure 1:**
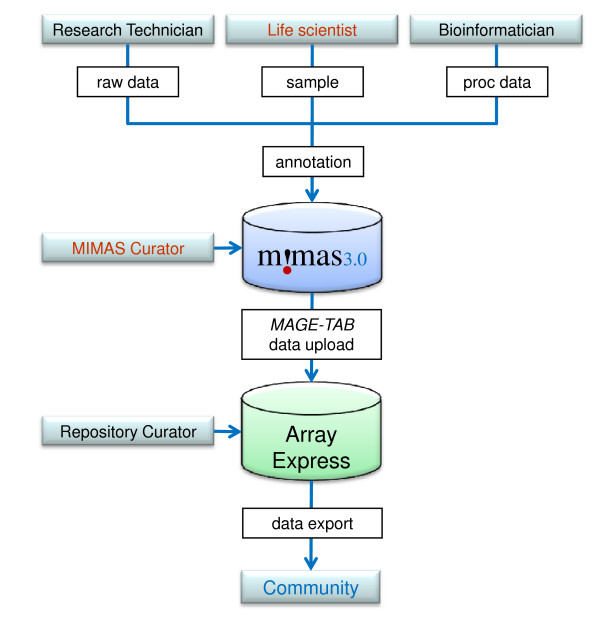
**MIMAS 3.0 Data Annotation and Submission Workflow**. Tasks performed by different types of users and curators are depicted. A single user can have several roles, depending on his or her technical skills and the organization of the facility or the consortium. The order in which files and annotation data are stored in MIMAS is flexible and organized within user-defined experiments. Raw data are unprocessed CEL (Affymetrix), tabular files and GFF3 files for DNA sequencing (Illumina). Proc data are processed data files. Sample is biomedical input describing the cells and tissues examined.

MIMAS 3.0 GUI is optimized to upload, annotate and manage microarray and UHTS experiments and it includes functionalities to search, download and export data. Its work-flow is designed to allow biological and biomedical researchers with little or no bioinformatics skills to input the data. This facilitates the task of high-through put data annotation often carried out by research technicians who work at service platforms that are accessible for large numbers of laboratories. It also provides the necessary infrastructure for annotation of biological samples and technical parameters that must follow experimental work to ensure proper data archiving (Figure 1). MIMAS 3.0 does not require any particular operating system, web browser or plug-in software.

### New Experiment annotation and management procedure

As described previously [25], MIMAS facilitates organization of samples according to experimental conditions and allows for propagation of annotation information among samples to speed up the process of data description. The intuitive user interface and the design allowing for input from different experts on the same project encourages interaction between technicians and experimental biologists during the annotation process (Figure 1). Another important novel feature is that files and annotation information may be entered into the system in any order, thus encouraging users to process data early on during the experimental work-flow. This is crucial especially for large projects that involve hybridization or sequencing of numerous samples over long periods of time which requires sustained data input to avoid data loss or incorrect annotation.

Several users may be involved at different stages of the annotation process. Since this increases the possibility of errors the data input is validated by a local MIMAS curator prior to uploading into the database. Therefore only curated information is available for data export. Moreover, the annotation data are verified a second time by curators at the EBI's ArrayExpress which further increases the reliability of data archiving. In this context we note that seamless exporting of data to ArrayExpress enables users to exploit its extensive Application Programmer Interface (API) for further programmatic access; we currently favor this generic approach over developing a MIMAS-specific API.

### Integration of new high-throughput methods and data formats

MIMAS contains parser modules for the file formats listed in Table 1. Extending our software solution to support new technologies and data file formats is a straightforward process. The list of supported technologies can be edited via the web interface. Integration of a new data format requires changes to the master parser Perl module that determines a file's format based on its name or its content. It is recommended, but not essential, to write a file-format-specific Perl module for verification of a file's integrity and validity and to extract certain data fields.

**Table 1:** Technologies and data formats supported by MIMAS 3.0.

**Technology**	**Raw data format**	**Processed data format**
Affymetrix GeneChips	Affymetrix CEL file (GCOS versions 3 or 4 or CCG version 1)	Tabular (Affymetrix MAS/GCOS versions 4 or 5, GEO MAS, GEO Series Matrix)
		
Affymetrix All-Exon Arrays		
		
Affymetrix Tiling Arrays		
		
Affymetrix Promoter arrays		
		
Affymetrix SNP arrays		
		
Affymetrix Gene Arrays		

Illumina BeadArrays	Tabular (Illumina BeadStudio ArrayExpress-format output plug-in)	
		
Illumina miRNA arrays		

Illumina Genome Analyzer		GFF 3

## Utility

MIMAS has been instrumental for managing and publishing high-throughput microarray data from many laboratories organized within the Swiss Array Consortium, including our own [29-32] and it is now also hosted by the bioinformatics platform [33] of Ouest-Genopole [34]. Its user-friendly interface enables life scientists to locally annotate and upload their raw GeneChip, BeadArray as well as UHTS data to certified repositories. We propose a flexible and scalable solution developed within a network of collaborators that includes bioinformatics experts, research technicians and life scientists. MIMAS is an integral part of our research program and as such it is a sustained software system. This is not to be taken for granted in the fast-paced bioinformatics field of data management solutions that have to deal with rapidly evolving and quickly emerging genome biological technologies.

## Discussion

MIMAS 3.0 is a versatile data management solution for the output of microarray experiments based on GeneChips and BeadArrays and data produced with ultra high-throughput DNA sequencing equipment. We do not associate raw data with UHTS experiments because their huge volume (up to 100 gigabytes) and the very large number of text and image files (up to 2000 text files and 14000 image files) produced by them makes storage and access via a web interface impractical. Moreover, no convention exists yet that defines what is considered to be useful raw data such as for example images, fluorescence intensity files, intermediate files or final filtered sequence lists. We therefore decided for the time being to support files in the GFF format which are generated by mapping DNA sequence read output to a reference genome.

The database design facilitates incorporation of novel data formats used in emerging technologies. The system is optimized for a multi-platform and multi-user environment with an up-to-date annotation interface enabling scientists and research technicians to efficiently process large quantities of genome biological data. As such, MIMAS 3.0 is unique among comparable data management software solutions that are often limited to a specific technical platform or that require complicated annotation procedures.

### Comparison with other solutions

Most of the alternative data management software solutions described below provide data export options in MAGE-ML format. This allows for easy data submission to the ArrayExpress and GEO public repositories. However, the MAGE-ML format is complex and has not evolved since 2002. In contrast, MIMAS supports the MAGE-TAB format which is editable in any spreadsheet software. It is therefore straightforward to review annotation in tabular form prior to submission, and to include additional information beyond the scope of the data management software. This could include for example geographical coordinates of locations where metagenomics samples were collected [35].

BASE (BioArray Software Environment) is a web based system under active development which accommodates data from high-density oligonucleotide microarrays (Affymetrix), BeadArrays (Illumina) and microarrays based on adhesion of DNA fragments onto a glass support from academic or commercial sources (two-color microarrays) [36] (we refer only to the website and not the original publication since version two of the system has been completely rewritten and is as yet unpublished). This software enables users to import and export experimental data in the Tab2MAGE format. However, the current version 2.9 provides only limited support for tiling or SNP arrays and lacks a solution for storing UHTS data. In our opinion this product implements a time consuming, repetitive, and thus error prone annotation procedure (Table 2). MARS (Microarray Analysis and Retrieval System) is a MIAME-compliant software suite for storing, retrieving, and analyzing multi color microarray but not GeneChip and BeadArray or UHTS data [37]. SBEAMS is a very elaborate system that supports a wide array of functional genomics technologies but does not support exporting in the MAGE-ML or MAGE-TAB formats. Moreover, the complexity of its interface makes it cumbersome to use for experimental biologist, biomedical researchers and clinicians [38]. MiMiR is designed to be used in clinical trials and provides an advanced security infrastructure for that purpose. As such, it provides specialized vocabularies embedded within a complex mapping tool [39]. The maxdLoad2 software has not been extended for several years and is a stand-alone solution installed in individual PCs. It does not support a server installation, which prevents collaboration and could lead to problems with data integrity [40]. MIAMExpress is practical especially for researchers who do not have a local data repository and wish to submit their data directly to ArrayExpress. However, it only supports a limited range of microarray platforms and no DNA sequencing platforms [41].

**Table 2:** Comparison of MIMAS 3.0 with other solutions as described in the literature.

	MIMAS 3	BASE 2	MARS	SBEAMS	MiMiR	maxdload2	MIAMExpress
Affymetrix GeneChip	*	*		*	*	*	*

Two-color Microarray		*	*	*	*	*	*

Illumina BeadArray	*	*					

UHTS	*						

MIAME compliant	*	*	*	*	*	*	*

MAGE-TAB export	*	*					*

MAGE-ML export		*	*		*	*	*

MGED Ontology support	*		*		*	*	*

Connectivity to analysis and visualization tools	*	*	*	*	*	*	

User and group management	*	*	*	*	*		

Object sharing and permissions management	*	*	*	*	*		

Web application	*	*	*	*			*

User Notifications/Messages	*	*	*				

Wizard-based annotation and experiment creation	*	*					

### Future work

We intend to maintain and further develop MIMAS 3.0 as a data annotation and archiving solution for technologies that yield information on DNA integrity, gene expression and protein-DNA interactions via microarrays and UHTS methods. Moreover, we wish to include other data types including the output of experiments aiming at RNA and protein expression by *in situ *hybridization and immunohistocytochemistry [42] and Tissue Microarrays (TMAs) that yield data and images for hundreds of normal and cancerous samples [43]. Finally, we ultimately plan to incorporate the output of protein expression data measured by mass spectroscopy into MIMAS [44].

## Conclusion

MIMAS 3.0 was developed by a network of software developers, computer scientists, life scientists, and research technicians involved in high-throughput data production, analysis and interpretation. In our experience such a constellation spawns solutions that are user-friendly, efficient and durable. A key problem of open source software is often, apart from programming errors, lack of long-term support. Our software has been successfully used for several years by a large number of laboratories in Switzerland and it was recently also set up at the bioinformatics platform of Biogenouest in France. MIMAS 3.0 is an important element of our ongoing genome biological research and will therefore continue to be developed in the foreseeable future. The software is freely available at the Sourceforge repository.

## Availability and requirements

• Project home pages: ; ; download via 

• Operating system(s): Linux/UNIX, Mac OS X, Windows

• Programming languages: Perl, SQL, JavaScript

• Apache web server with mod_perl extension

• Oracle 9 or later or MySQL 4.1 or later.

## Abbreviations

API: (Application Programmer Interface); GCOS: (GeneChip Operating System); GFF3: (Generic Feature Format Version 3); GUI: (Graphical User Interface); MAGE: (MicroArray and Gene Expression); MAS: (Microarray Analysis Suite); MINSEQE: (Minimum Information about a high-throughput Nucleotide SeQuencing Experiment); MGED: (Microarray Gene Expression Data); MIAME: (Minimal Information about a Microarray Experiment); UHTS: (Ultra high-throughput DNA Sequencing); TMAs: (Tissue Microarrays).

## Authors' contributions

AG and LH designed and developed the database software and AG also contributed to the manuscript. IX and JR contributed to software development and they host the database in Lausanne. RL maintains and curates the database in Lausanne. OC maintains the database in Rennes. MP contributed to the design and wrote the paper. All authors read and approved the final manuscript.
